# Relationship between immune gene signatures and clinical response to PD-1 blockade with pembrolizumab (MK-3475) in patients with advanced solid tumors

**DOI:** 10.1186/2051-1426-3-S2-P80

**Published:** 2015-11-04

**Authors:** Mark Ayers, Jared Lunceford, Michael Nebozhyn, Erin Murphy, Andrey Loboda, Andrew Albright, Jonathan Cheng, S Peter Kang, Scot Ebbinghaus, Jennifer Yearley, Veena Shankaran, Tanguy Seiwert, Antoni Ribas, Terri McClanahan

**Affiliations:** 1 Merck & Co., Inc., Kenilworth, NJ, USA; 2 Merck & Co., Inc., Palo Alto, CA, USA; 3 University of Washington, Seattle, WA, USA; 4 The University of Chicago, Chicago, IL, USA; 5 University of California at Los Angeles Medical Center, Los Angeles, CA, USA

## Background

Immune checkpoint inhibition with anti–PD-1 monoclonal antibodies such as pembrolizumab has demonstrated robust, durable anti-tumor activity against many advanced malignancies. We analyzed immune-related gene expression profiles in pembrolizumab-treated patients with advanced solid tumors to identify immune gene signatures correlated with clinical benefit.

## Methods

RNA was extracted from formalin-fixed, paraffin-embedded sections of baseline tumor samples and analyzed using a custom 680-gene set on the NanoString nCounter platform. A 10-gene preliminary “interferon-gamma” (IFN-γ) signature was developed in a discovery set of 19 patients with melanoma treated with pembrolizumab in the Phase 1b KEYNOTE-001 study (NCT01295827) and was later complemented with a 28-gene preliminary “expanded immune” signature. These 2 signatures were subsequently tested and refined in an independent cohort of 62 additional patients with melanoma treated in KEYNOTE-001. Further evaluation of the refined signatures was performed in 43 patients with head and neck squamous cell carcinoma (HNSCC) and 33 patients with gastric cancer enrolled in the Phase 1b KEYNOTE-012 study (NCT01848834).

## Results

In the melanoma validation set, the IFN-γ and expanded immune signatures were significantly correlated with ORR (*P*=0.047 and 0.027) and PFS (*P*=0.016 and 0.015). The IFN-γ signature was refined from 10 genes to 6, and the expanded immune signature from 28 genes to 18. Two new signatures enriched in T cell markers and MHC class I and II genes were enumerated based on analysis of top-ranked genes on the platform in melanoma samples: “TCR signaling” (13 genes) and “de novo” (33 genes). All signatures were independently tested in HNSCC and gastric cancer and found to be significantly correlated with clinical benefit (Table 1). Tumors lacking an immune phenotype, as suggested by low values of signature scores, did not respond to pembrolizumab. Some non-responders had scores similar to those of responders, suggesting an immune phenotype is necessary but not sufficient for response.

**Table 1:** Nominal 1-sided *P* values for ORR and PFS calculated from logistic and Cox regression, respectively, using signature score as a continuous variable.

Signature	HNSCC		Gastric Cancer	
	**ORR**	**PFS**	**ORR**	**PFS**

IFN-λ	0.005	<0.001	0.077	0.032

TCR signaling	0.071	0.002	0.034	0.024

Expanded immune	0.015	<0.001	0.062	0.049

De novo	0.018	<0.001	0.068	0.037

## Conclusions

Immune-related gene expression signatures composed of genes associated with T cell cytotoxic function, antigen presentation machinery, and IFN-γ signaling represent reproducible and sensitive tools that define common features of the immune microenvironment associated with response to pembrolizumab across multiple tumor types.

